# Fundamental research progress of mild hypothermia in cerebral protection

**DOI:** 10.1186/2193-1801-2-306

**Published:** 2013-07-07

**Authors:** Long Bao, Feng Xu

**Affiliations:** Department of Emergency medicine, The First Affiliated Hospital of Soochow University, Suzhou, 215006 China

**Keywords:** Mild hypothermia, Neuroprotection, Inflammatory cytokines, Neurotransmitter, Apoptosis

## Abstract

Through the years, the clinical application of mild hypothermia has been carried out worldwide and is built from the exploration and cognition of neuroprotection mechanisms by hypothermia. However, within the last decade, extensive and fundamental researches in this area have been conducted. In addition to aspects of the previous findings, scholars have discovered several new contents and uncertain results. This article reviews and summarizes this decade’s progression of mild hypothermia in lowering the cerebral oxygen metabolism, protecting the blood–brain-barrier, regulating the inflammatory response, regulating the excessive release of neurotransmitters, inhibiting calcium overload, and reducing neuronal apoptosis. In many aspects, particularly in regulating inflammatory reverse reaction, various results have been reported and therefore guide scholars to conduct more detailed analysis and investigation in order to discover the inherent theories surrounding the effect of mild hypothermia, and for better clinical services.

## Introduction

Cold and humanity have a long-standing relationship. For the past twenty years, people have begun to recognize the protective effects of mild hypothermia on the body, especially on the brain. Therapeutic hypothermia has been extensively studied in the laboratory. It is one of the most robust neuroprotectants studied to date, and recent clinical studies have established a role for therapeutic hypothermia in neuroprotection in some clinical conditions, including canoxic brain injury due to cardiac arrest (Bernard et al. 2002), hypoxic ischaemic neonatal encephalopathy (Gluckman et al. 2005; Shankaran et al. 2005), stroke (Wu and Grotta 2013), brain (Dietrich and Bramlett 2010) and spinal cord trauma (Dietrich et al. 2009). The specific mechanisms of this protection remain unclear. It affects nearly every metabolic, molecular and cellular event in cell death to promote tissue preservation (Dine and Abella 2009).

This review focuses on the development of fundamental research on mild hypothermia neuroprotection over the past decade. It summarizes various mechanisms by which hypothermia may be protective including reduced cerebral metabolism, blood–brain barrier permeability, excitatory amino acids as well as inflammatory events, inhibiting calcium overload, and apoptotic pathways.

## Review

### Lowering of cerebral metabolic rate of oxygen (CMR_O2_) and subsequent energy depletion, reducing the accumulation of lactic acid

An important mechanism for the neuroprotective effects of hypothermia is a reduction or delay in metabolic consumption during the stress of a CNS injury (Dietrich et al. 2009). The traditional view is that hypothermia reduces CMR_O2_ of approximately 5% per degree Celsius (Erecinska et al. 2003). In 2008, German scholars reported that using mild therapeutic hypothermia in severe traumatic brain injury patients, one-degree drops in temperature led to a 5.9% reduction in energy. And there was a close linear correlation between body temperature and basal metabolism (Saur et al. 2008). Hypothermia lower metabolic and energy demands which can have beneficial effects on cytoplasmic ATP stores and the maintenance of normal transmembrane ion and neurotransmitter gradients. By limiting the consumption of oxygen and glucose by the brain, hypothermia reduces the risk of energy failure (Choi et al. 2012).

Under normal conditions, cerebral blood flow (CBF) to the brain is apporoximately 50 ml/100 g/min. Hypothermia decreased CBF from 48 ml/100 g/min in normothermic animals to 21 and 11 ml/100 g/min at 33°C and 29°C, respectively (Liu and Yenari 2007). The appearance of Positron Emission Computed Tomography (PET) imaging makes CMR_O2_ and CBF examination become more visualized. Japanese scholars reported that in mild therapeutic hypothermia of patients with ruptured aneurysm, PET revealed a decrease in CMR_O2_ and CBF significantly, on both ipsilateral and contralateral side, up to 1/3-1/2 (Kawamura et al. 2000).

After brain injury, anaerobic metabolite lactate level rises due to various causes of inadequate cerebral oxygen supply. By preserving the brain’s metabolic stores, hypothermia can prevent the downstream consequences of increased lactate production and the development of acidosis. Moreover, mild therapeutic hypothermia can slow down the elevation of lactic acid in the cerebrospinal fluid and brain microdialysate, and even stop the rise (Jiang et al. 2004). It is currently felt that, although cerebral hypothermia may not prevent the eventual depletion of ATP or lactate accumulation during a prolonged period of ischemia, hypothermia could certainly slow ATP depletion during a brief ischemia period.

The mechanism of how mild hypothermia could reduce CMR_O2_ is still unclear. Recent studies have shown that mild hypothermia and anesthesia can both reduce cerebral oxygen metabolism safely (Ouchi et al. 2006). But maybe they have different mechanisms. One study found that (Michenfelder 2002), constant infusion of anesthetic initially produced the expected progressive decrease in CMR_O2_ until a mean decrease to about 58%. Thereafter, despite continued administration of anesthetic, no further effect on CMR_O2_ was observed. Anesthetics, mainly by reducing cerebral electrical activity to decrease CMR_O2_, revealed no alteration in normal cerebral metabolic pathways, hence it would provide no cerebral protection during hypoxia. In another study, German scholars observed the effect of mild hypothermia on CMR_O2_ and cerebral function through an increase in intracranial pressure (ICP), and subsequent decrease in cerebral perfusion pressure (CPP) to reduce CBF (Bauer et al. 2000). This study found that mild hypothermia improved cerebral oxygen balance by reduction of brain energy demand. The brain electrical activity was less suppressed under hypothermia during severe blood flow reduction, which indicated that hypothermic neuroprotection may involve some other mechanisms. And further studies are needed to make it clear.

While mild hypothermia is reducing the brain metabolisms, there are no negative effects to the body. Low temperature decreases brain metabolism and reduces oxygen demand. But this is not at the expense of other organ or system, therefore it does no harm to the body. For example, hypothermia does not impair the compensatory cardiovascular responses of the fetus to acute moderate hypoxia (Chihara et al. 2003). Nevertheless, mild hypothermia cannot always be beneficial. One suggested that severe traumatic brain injury patients with very low CBF and CMR_O2_ (CBF < 28.9 ml/100 g/min,CMR_O2_ < 1.17 ml/100 g /min) are not suitable for mild therapeutic hypothermia (Masaoka 2010).

### Prevention of blood–brain-barrier (BBB) disruption and subsequent amelioration of cerebral edema

Cerebral edema formation after a period of brain injury is mostly the result of change in BBB permeability. BBB disruption after brain injuries is caused by structural and functional impairment of components of the neurovascular unit, including tight-junction proteins, transport proteins, basement membrane, endothelial cells, astrocytes, pericytes and neurons. Models of brain ischemia, trauma and intracerebral hemorrhage have shown that mild to moderate hypothermia protects the BBB and prevents edema formation (Preston and Webster 2004; MacLellan et al. 2006; Kawanishi et al. 2008; Oda et al. 2011). This might explain the effectiveness of mild induced hypothermia in reducing intracranial pressure (ICP) after traumatic brain injury (TBI) (Sadaka and Veremakis 2012). In the recent years, scholars have attempted to explain the protection mechanism.

Hypothermia prevents the activation of proteases responsible for degrading the extracellular matrix, such as the Matrix metalloproteinases (MMPs) (Lee et al. 2005; Truettner et al. 2005; Nagel et al. 2008). MMPs have been implicated in blood–brain barrier disruption because they can degrade the extracellular matrix. Mild hypothermia attenuates blood–brain barrier disruption, decreases MMP expression, and suppresses MMP activity. Hypothermia also attenuates edema formation by preserving the brain’s water balance. Aquaporins are a family of water channel proteins that control the movement of water across cell membranes. Mild hypothermia can significantly reduce the over-expression of Aquaporin 4 (AQP-4) and protect the BBB, thereby reducing cerebral edema (Dai et al. 2006).

For the time window of cooling beginning, even when hypothermia is applied twenty-four hours after brain injury, It can significantly reduce the cerebral edema formation. And the neuroprotective mechanisms include reducing BBB disruption (Kawanishi et al. 2008).

### Effects on inflammatory mediators

Inflammation is an essential tool to defend oneself against infectious organisms. However, it becomes detrimental when it is prolonged or attacks self antigens. (Simi et al. 2007) After brain injury, pro- and anti-inflammatory cytokines are quickly and extensively upregulated (Huang et al. 2006; Nilupul et al. 2006; Wang et al. 2007). Pro-inflammatory cytokines stimulate and aggravate the inflammatory response. The most prominent cytokines after brain injury are interleukin-1β (IL-1β), tumor necrosis factor-α (TNF-α),and IL-6 (Hossmann 2006; Lai and Todd 2006). On the other hand, anti-inflammatory cytokines inhibit the expression of pro-inflammatory cytokines and reduce inflammation. Transforming growth factor-β (TGF-β) and IL-10 belong to this category and are most studied (Vitkovic et al. 2001). However, cytokines can not unequivocally be divided into pro- or anti-inflammatory cytokines and may exert neurotoxic as well as neuroprotective effects (Sriram and O'Callaghan 2007; Kadhim et al. 2008). The balance between deleterious and beneficial effects of cytokines will depend on the physiological and biochemical context in the brain (Ceulemans et al. 2010).

For example, TNF-α released in the striatum is considered to cause neurodegeneration, while release in the hippocampus could promote neuroprotection. One suggests that the detrimental effects occur in the early phase of the inflammatory response and the more beneficial effects in a later stage (Amantea et al. 2009). Another suggests that soluble TNF-α (which binds to TNF receptor 1) would cause primarily detrimental effects whereas membrane bound TNF-α (which binds to TNF receptor 2) would signal for neuroprotection (Fontaine et al. 2002). Other studies suggest that TNF-α can also be neuroprotective by acting through TNF receptor 1 (McCoy and Tansey 2008; Lambertsen et al. 2009). In conclusion, neurotoxic or neuroprotective effects will depend on several factors such as the extent of microglial activation in specific brain regions, timing and threshold of TNF-α expression and of its receptors and on the conditions that stimulate TNF-α signaling.

After the application of mild therapeutic hypothermia, these inflammatory factors are either up-regulated or down-regulated. The role of mild hypothermia as pro-inflammatory or anti-inflammatory remains unclear. In recent years, all study results vary widely, including *in vitro*, zoology, and clinical trials. Studies on human peripheral blood mononuclear cell *in vitro* show that mild hypothermia affects the balance of cytokines produced by monocytes, leading to a pro-inflammatory state (Matsui et al. 2004; Matsui et al. 2006; Rosengren et al. 2007). It appears to be involved in the immune alterations, reduce the host defense reaction, and thereby increase the chance of infection. Results from Zoology studies (Kentner et al. 2002; Hofstetter et al. 2007; Huet et al. 2007) suggest that mild hypothermia attenuates the inflammatory response *in vivo and* increase anti-inflammatory factor, thus contributing to its beneficial role in neuroprotection. Additionally, mild hypothermia reduces mortality during endotoxemia in animal models (Huet et al. 2007). The results of clinical trial report that mild hypothermia does not affect the level of inflammatory factors (Horan et al. 2004).

It is considered that the wide-ranged outcomes on the role of mild hypothermia on immune may be attributed to the numerous kinds of animal model, different types of brain injury, and the depth and duration of mild hypothermia, as well as the dual role of inflammation and cytokines in brain injury. The precise roles of the immunoinflammatory reactions in the dynamics of brain injury and repair are still unclear. Thus, activated cells and their products could have detrimental effects leading to secondary brain damage (Lucas et al. 2006), certain molecular pathways that are triggered during these immunoinflammatory reactions are somehow also involved in reparative processes (Correale and Villa 2004) and could have different or even opposing effects. These phenomena require further clarification.

### Inhibition of excitotoxic neurotransmitter release

A well-known mechanism by which hypothermia exerts neuroprotection is by reducing release of excitoxic neurotransmitters after all kinds of brain injuries, potentially leading to secondary brain injury (Urbano and Oddo 2012). Among variety of brain neurotransmitters, excitatory amino acid (EAA), and nitric oxide (NO) have been most focused on in recent years.

### Excitatory amino acid (EAA)

Excitatory amino acids, including glutamate and aspartate, are significantly elevated after ischemia, hypoxia, trauma, and poisoning. Activation of the corresponding excitoxic receptor is of the important factors resulting in secondary injury to the brain. EAA concentration after an acute head injury is associated with the degree of brain damage.

More than twenty years ago, intraischemic hypothermia (33°C and 30°C) was reported to attenuate the rise in extracellilar levels of striatal glutamate and dopamine after global ischemia (Busto et al. 1989). Recent study has made the result even more intuitive. After being exposed to eight minutes of asphyxiation, the extracellular glutamate and dopamine levels increase to 30 times and 50 times, respectively. However, under mild hypothermia, the glutamate and dopamine levels are not significantly change (Hachimi-Idrissi et al. 2004).

Prevention of the accumulation or release of glutamate by hypothermia may be attributed to the effect of cooling on metabolism, which preserves tissue ATP levels. ATP is needed to maintain ion gradients, and when these concentration gradients are disturbed, calcium influx occurs and leads to increased extracellular glutamate levels (Yenari and Han 2012). The glutamatergic receptors, AMPA and NMDA, are also modulated by hypothermia (Friedman et al. 2001). Hypothermia may prevent the consequences of excitotoxicity by limiting calcium influx through AMPA channels. The glutamate receptor 2 (GluR2) subunit of the AMPA receptor is thought to limit calcium influx, and its downregulation by brain injury may lead to the entry of excess calcium. One study demonstrated that hypothermia attenuates ischemia-induced downregulation of GluR2 in a model of global cerebral ischemia (Colbourne et al. 2003).

Recent study has revealed that, elevated level of glutamate after cerebral ischemia is not due to an increase in release, but rather an obstruction in re-uptake. However, mild hypothermia can increase the re-uptake and consequently reduce the level of glutamate (Asai et al. 2000).

There should be a balance between excitatory amino acids and inhibitory amino acids after brain injury. Mild hypothermia effectively decreases the damage of the cerebral tissue by reduction of glycerin and excitatory amino acids release (Prandini et al. 2005), increases the inhibitory amino acid gamma-aminobutyric acid (GABA) (Zhang et al. 2008) to protect neurons. Inhibitory amino acids antagonizes the effects of excitatory amino acids, and mild hypothermia has a clear correlation between them.

Studies have shown that the inhibition effect of mild hypothermia on EAAs release in penumbra area and non-injury area is more significant. However, there is no significant results in the irreversible damage (core) area. For the time window of cooling beginning, One suggests that mild hypothermia should be applied immediately after ischemia in order to obtain better neuroprotection. Post- ischemic mild hypothermia can significantly suppress the excessive release of EAAs from the very beginning (Zhang et al. 2008).

### Nitric oxide (NO)

Oxidative stress can damage the organism if the physiological balance between oxidants and anti-oxidants is disrupted in favor of the former. The key radical after brain injury is superoxide anion, produced by xanthine oxidase and NADPH oxidase. L-arginin is transformed into nitric oxide (NO) via 3 types of NO synthases (NOS): neuronal, endothelial and inducible (n-, e-, iNOS respectively). These NOS are increased after brain ischemia (Lakhan et al. 2009).

Under mild hypothermia, changes of NO and NOS are important mechanisms for the protection of neurons. The protective effects are proved in animal models of cerebral ischemia, cerebral hemorrhage, traumatic brain injury, and SAH. NO accumulates immediately after injury, while NOS activity in damaged brain increases. Mild hypothermia can reduce the level of NO and suppress the activity of NOS, hence protect the neurons. NO plays an important role in the occurrence and development of post-injury cerebral edema. Mild hypothermia can lower the NO levels in internal jugular vein significantly, then reduce the degree of cerebral edema. Subsequently it can alleviate the secondary cerebral injury, and reduce mortality (Ceulemans et al. 2010). Conversely, some studies show different results. One suggests that hypothermia do not affect NO production as compared to the normothermia, using cultured monocytes with lipopolysaccharide *in vitro* (Matsui et al. 2006)*.*

In recent years, scholars have begun to study the 3 types of NOS. Influence of mild hypothermia during intraischemic and postischemic mild hypothermia were different. Intraischemic hypothermia effect was strong on iNOS expressions, whereas postischemic hypothermia effection was strong on nNOS expressions (Karabiyikoglu et al. 2003). Other scholars found that mild hypothermia did not change the expression of nNOS, but it significantly induced the attenuation of nNOS activity. This effect could be one of the neuroprotective mechanisms of hypothermia (Hayashi et al. 2011).

Mild hypothermia can inhibit the expressions of NOS in cortical penumbra (IP), and reduce NO and its metabolites, which is similar to its effect on excitotoxic neurotransmitters, thereby play a neuroprotective role (Van Hemelrijck et al. 2005). The difference is that mild hypothermia applied on brain injury has certain degree of inhibition of NO in the core region of injuty. For the time window of cooling beginning, one suggests that mild hypothermia exerts time-dependent effects on lowering the level of iNOS; delayed mild therapeutic hypothermia could also play a neuroprotective role (Seo et al. 2012).

The relationship between the complex neurotransmitters in the brain is mutually influential. Elevated level of NO might just be one part of the many other neurotransmitter channels. Elevated level of glutamate in the cerebral cortex can increase the level of extracellular NO and its metabolites (nitrite and nitrate), but mild hypothermia can inhibit this process. The effect of inhibition of iNOS by mild hypothermia might just be a part of nuclear factor kappa B (NF-κB) inhibition. Following cerebral ischemia, NF-κB activation leads to the expression of many inflammatory genes involved in the pathogenesis of stroke. Mild hypothermia prevents nuclear NF-κB translocation and DNA binding by inhibiting the activity of inhibitor of NF-κB kinase (IKK). IKK is required for the phosphorylation and degradation of NF-κB inhibitor (IκB), thereby allowing NF-κB to enter the nucleus, where it can upregulate target genes ,such as iNOS and TNF-α (Han et al. 2003; Yenari and Han 2006). Cerebral ischemia induces the activation of calcium/calmodulin kinase II (CaM-KII), which modulates many enzymes. Under the influence of mild hypothermia, CaM-KII attenuates nNOS activity, is perhaps one of the neuroprotective mechanisms (Hayashi et al. 2011).

### Reducing Ca^2+^ influx and the toxicity effect of Ca^2+^ on neurons

Calcium plays an important role in the normal physiological state, as well as in many pathological conditions of cell damage processes. Excessive Ca^2+^ in the cells can initiate the endogenous destruction mechanisms that damage the cells. Currently, some people consider calcium as the “last common channel” leading to cell death in a variety of pathological states. Many animal and clinical trials have confirmed that, after a variety kinds of brain injuries, the neuronal intracellular Ca^2+^ will quickly overload, and it is partly because of the release of mitochondrial calcium stores, which causes impairment of the neuron structure and function (Moquin and Chan 2010). Ca^2+^ overload is also involved in the pathogenesis of epilepsy. CA3 area of hippocampus plays a major role in Ca^2+^ overload pathogenesis of epilepsy (Zheng et al. 2013). Mild hypothermia can reduce Ca^2+^ influx, power the declined Ca^2+^-ATPase activity after brain injury, restore mitochondrial energy, thereby stabilize mitochondrial function of the calcium storage, regulate synaptic function, then block the toxic effects of calcium on neurons. In recent years, *in vitro* experiments have established the same result. Scholars cultured hippocampal slice under the condition of oxygen and glucose deprivation, and got the conclusion that mild hypothermia could reduce glutamate and Ca^2+^ release, thus increase neuronal survival (Feiner et al. 2005).

Nowadays, the effect of mild hypothermia on some important calcium-related protein has been gradually concerned. Calcium protease (Calpain) is a calcium-dependent protease which can be activated by Ca^2+^*In vivo* and exhibit the activity of proteolytic enzyme. Its main targets are cytoskeletal proteins, protein kinases, phosphatases, and hormone receptors. After brain injury, the neuroprotective mechanisms of mild hypothermia might be related to the significant suppression of Calpain level after transcription (Haranishi et al. 2005). People have found that intraneuronal calpain activity changes following brain injury are involved in the pathological process of cellular injury. Mild hypothermia might inhibit this activity changes by suppressing the activity of CalpainII, and reducing the degradation of the cytoskeleton, thus play neuroprotective role (Liebetrau et al. 2004; Sui et al. 2007).

In recent years, calcium-sensing receptor (CaSR) has been public concerned. The receptor is particularly sensitive to extracellular calcium concentration. It may regulate Ca^2+^ influx and intracellular Ca^2+^ release, causing the extracellular Ca^2+^ level to change rapidly. One demonstrated that after ischemic brain injury, CaSR expression increased and GABA-B-R1 expression decreased, nevertheless this was reversible by mild hypothermia (Kim et al. 2011).

### Effects on cell death pathways

Mild hypothermia has been shown to affect several aspects of apoptotic cell death. It decreases *cysteinyl aspartate specific proteinase* (Caspase)-3 mRNA expression in caspase-dependent pathway (Lotocki et al. 2006), apoptosis inducing factor (AIF) mRNA expression in caspase-independent pathway (Zhao et al. 2007; Robertson et al. 2009), and Fas mRNA expression in extrinsic pathway (Yenari and Han 2012). Hypothermia inhibits apoptosis after cerebral ischemia/reperfusion through multiple ways, thereby plays a neuroprotective role.

Mild hypothermia can interfere with the intrinsic payhway by changing the expression of BCL-2 family members, reducing cytochrome c release (Yenari et al. 2002) and decreasing caspase activation (Liu and Yenari 2007). In models of global cerebral ischemia, hypothermia leads to reduction in pro-apoptotic BCL-2 family member such as BCL-2 associated X (BAX) and increases in the anti-apoptotic member BCL-2 (Drury et al. 2010). Acting downstream of BCL-2 family proteins, protein kinase Cδ (PKCδ) is a PKC isoform that has been shown to contribute to ischemia injury (Bright et al. 2004), and caspase 3 leads to translocation of PKCδ from cytosol to the mitochondria and to the nucleus to induce apoptosis (Raval et al. 2005). By contrast, a different isoform, PKCϵ is anti-apoptotic, and is degraded by caspases. Interestingly, hypothermia does not seem to alter overall levels of PKCδ (Lee et al. 2009), but in blocks ots translocation to the mitochondria and the nucleus and stimulates the action of PKCϵ after ischemia.

The extrinsic apoptotic pathway also seems to be activated in brain injury. The most widely studied apoptosis-inducing receptor and ligand in this pathway are FAS and FASL (Rosenbaum et al. 2000). Mild hypothermia seems to suppress the expression of both proteins in models in which neuroprotection is observed (Liu et al. 2008).

As partly mentioned above, hypothermia has anti-apoptotic effects that could also be mediated by affecting NF-κB (Van Hemelrijck et al. 2005). In normal conditions, NF-κB is present in the cytoplasm, but is bound to a family of inhibiting proteins. To be activated, IKK must phosphorylate these inhibiting proteins to liberate NF-κB and let it enter the nucleus and induce gene expression (Han et al. 2003). Hypothermia was able to reduce IKK expression, resulting in less NF-κB translocation (Webster et al. 2009). Inhibition of NF-κB activation could be key to the neuroprotective effects of hypothermia.

Electron microscopic observation has shown significant morphological damage in cerebellar cortex neuron after ischemia/reperfusion, chromatin condensation, margination, enhanced electron density, crescent-shaped or irregular shaped nuclear, smaller sized cell, concentrated cytoplasm and TUNEL staining showed significant Purkinje cell apoptosis. Structural damage of neuron under mild hypothermia is remarkably decreased; apoptotic cells and apoptosis rate are significantly reduced.

Studies have found that mild hypothermia can slow down the process of neuron apoptosis in cerebral ischemia-reperfusion. However, after re-warming the apoptotic speed rebounds rapidly. Therefore, the time window for mild hypothermia beginning should be actively sought (Tissier et al. 2011). One research found that pre-operation hypothermia could reduce the damage of neuron and glia in the reperfusion phase of ischemia/reperfusion brain injury (Yokobori et al. 2013). Therefore, hypothermia should be initiated as soon as possible to achieve its optimal beneficial effect.

MicroRNAs (miRNAs), a subset of non-coding RNAs, have been a topic of recent investigation in brain injury models, including stroke, where their expression increases as early as 2 h after ischemia onset (Rink and Khanna 2011). miRNAs are thought to play a part in silencing mRNAs, but they can also regulate a range of signaling pathways. It is conceivable that they have an important role in stroke pathogenesis, and the roles of specific miRNAs are currently under investigation. A recent report in a model of traumatic brain injury showed that cooling alters the expression of several miRNAs (Truettner et al. 2011). A few miRNAs, including miR-874 and miR-451, were most strongly affected. Cooling decreased the expression of both miRNAs at 7 h, but miR-451 was increased by cooling at 24 h compared to normothermia. Further research is needed to better define their role in brain injury.

## Conclusions

Mild hypothermia has been shown to be protective in various models of brain injury. Deeper insights into physiopathologic mechanisms implicated in the aggravation of brain injury and the causation of secondary brain damage might provide new means for secondary neuroprotection. Studies have provided the result of combined administration of calcium and glutamate antagonists (i.e. Mg^2+^), antioxidants (i.e. tirilazad), and mild hypothermia which offered superior neuroprotective effect compared with the custom treatment (mannitol, nimodipine, dexamethasone and phenobarbital) in an animal model subjected to cerebral ischemia (Scholler et al. 2004). However, this result requires further investigation. Certainly, it needs a very long time to be translated to clinical use.

In summary, mild hypothermia affects nearly every metabolic, molecular and cellular event in cell death to promote tissue preservation. Clinical application of mild therapeutic hypothermia is on the base of lab researches. It is clear that more research is still needed to understand its biological significance as well as how it can be applied in clinical conditions effectively. As studies are gradually revealing the mystery of mild hypothermia in neuroprotection, we believe that its clinical application will also become more advanced. Mild therapeutic hypothermia will be more brilliant in the next decade.

## Abbreviations

CMRO2: Cerebral metabolic rate of oxygen
CBF: Cerebral blood flow
PET: Positron emission computed tomography
ICP: Intracranial pressure
CPP: Cerebral perfusion pressure
BBB: Blood–brain-barrier
TBI: Traumatic brain injury
MMPs: Matrix metalloproteinases
AQP-4: Aquaporin 4
IL-1β: Interleukin-1β
TNF-α: Tumor necrosis factor-α
IL-6: Interleukin-6
TGF-β: Transforming growth factor-β
IL-10: Interleukin-10
EAA: Excitatory amino acid
NO: Nitric oxide
GABA: Gamma-aminobutyric acid
NOS: NO synthases
nNOS: Neuronal NOS
eNOS: Endothelial NOS
iNOS: Inducible NOS
NF-κB: Nuclear factor kappa B
IKK: Inhibitor of NF-κB kinase
IκB: NF-κB inhibitor
CaSR: Calcium-sensing receptor
Caspase: Cysteinyl aspartate specific proteinase
AIF: Apoptosis inducing factor
BAX: BCL-2 associated X
PKCδ: Protein kinase Cδ
miRNAs: MicroRNAs.
